# Supplementary material for: Assessment of self-rated health: The relative importance of physiological, mental, and socioeconomic factors
Source: PLoS One. 2022 Apr 18;17(4):e0267115. doi: 10.1371/journal.pone.0267115 (PMC9015117; doi:10.1371/journal.pone.0267115)
Supplement: S1 Appendix — (DOCX) [file pone.0267115.s001.docx]

**Appendix to the article What contributes to the assessment of Self-Rated Health?**

Figure A1: Distribution of biomarkers (CRP, Glucose, TG, HDL, LDL/HDL ratio), Qualitas 2016/2017 survey, N = 1 030


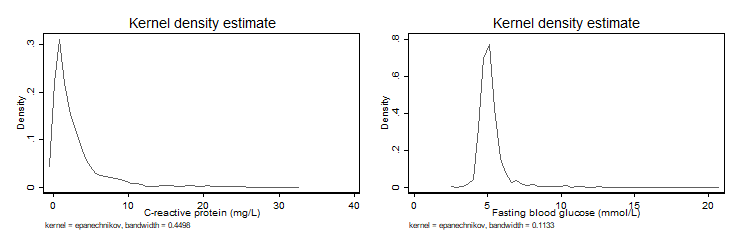


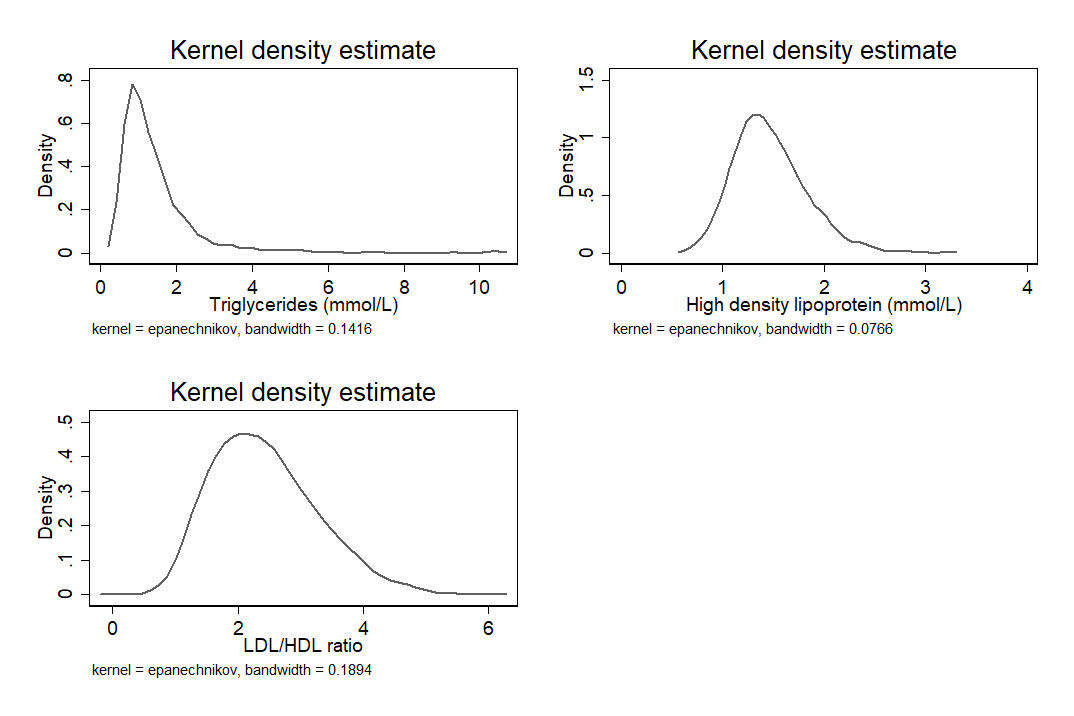


Table A1: Measurement model for mental health, Qualitas 2016/2017 survey, N=1 053

|  | Model 1 | | Model 2 | |
| --- | --- | --- | --- | --- |
|  | Coef | S.E. | Coef | S.E. |
| It1: You felt depress |  |  |  |  |
| Depress | 1 |  | 1 |  |
| Constant | 1.866** | -0.023 | 1.866** | -0.023 |
| It2: You felt that everything you did was an effort |  |  |  |  |
| Depress | 0.990** | -0.058 | 1.006** | -0.058 |
| Constant | 1.809** | -0.023 | 1.809** | -0.023 |
| It3: You felt sad |  |  |  |  |
| Depress | 0.814** | -0.043 | 0.830** | -0.043 |
| Constant | 1.712** | -0.022 | 1.712** | -0.022 |
| It4: You felt that you could not get going |  |  |  |  |
| Depress | 1.037** | -0.062 | 1.070** | -0.062 |
| Constant | 1.996** | -0.023 | 1.996** | -0.023 |
| Sleep quality |  |  |  |  |
| Depress |  |  | 0.576** | -0.055 |
| Constant |  |  | 1.996** | -0.024 |
| var(e.It1) | 0.283** | -0.019 | 0.293** | -0.018 |
| var(e.It2) | 0.289** | -0.018 | 0.290** | -0.018 |
| var(e.It3) | 0.339** | -0.019 | 0.338** | -0.018 |
| var(e.It4) | 0.248** | -0.018 | 0.240** | -0.017 |
| var(e.sleep) |  |  | 0.511** | -0.023 |
| var(Depress) | 0.280** | -0.026 | 0.270** | -0.025 |
| cov(e.It1,e.It3) | 0.095** | -0.015 | 0.099** | -0.014 |
| **FIT STATISTICS** |  |  |  |  |
| RMSEA | 0.039 |  | 0.038 |  |
| Probability RMSEA <= 0.05 | 0.506 |  | 0.709 |  |
| CFI | 0.999 |  | 0.996 |  |
| TLI | 0.993 |  | 0.989 |  |

Figure A2: Diagnostics for the regression analysis with dependent variable SRH and independent variables: age, sex, location, biomarkers

Figure A3: Diagnostics for the regression analysis with dependent variable SRH and independent variables: age, sex, location, marital status, education, economic activity, and income

Figure A3: Diagnostics for the regression analysis with dependent variable SRH and independent variables: age, sex, location, medication, number of health conditions, depressive symptoms, BMI, alcohol consumtpion, and smoking

**Self-rated health, socioeconomic characteristics, biomarkers, medication, self-reported health measures, mental health indicators, and health behavior**

Table A2 zooms in on socioeconomic characteristics. Model 1, serving as a baseline model, controlled for age, sex, and location. Age was the only variable that was significantly linked to SRH, and an age difference of 10 years was associated with a 0.2 shift in SRH. Sex and location (Prague vs. South Bohemia) were not significantly associated with SRH at the 0.05 significance level. However, importantly, these controls explained 16% of the total SRH variance.

**Table A2:** Results of the ordinary least squares (OLS) regression models with dependent variable SRH, displaying regression coefficients, standardized coefficients (beta), standard errors (in parentheses), and significance level. Source: QUALITAS 2016/2017 survey (N = 1021).

|  | M1 | | M2 | | M3 | | M4 | |
| --- | --- | --- | --- | --- | --- | --- | --- | --- |
|  | Coef. | Beta | Coef. | Beta | Coef. | Beta | Coef. | Beta |
| Age | -0.020** | -0.395** | -0.019** | -0.379** | -0.021** | -0.414** | -0.024** | -0.475** |
|  | -0.001 |  | -0.002 |  | -0.002 |  | -0.002 |  |
| Male | 0.019 | 0.011 | 0.047 | 0.029 | 0.000 | 0.000 | -0.081 | -0.048 |
|  | -0.047 |  | -0.047 |  | -0.048 |  | -0.054 |  |
| Location | 0.031 | 0.019 | -0.02 | -0.013 | -0.047 | -0.029 | -0.043 | -0.026 |
|  | -0.047 |  | -0.047 |  | -0.047 |  | -0.052 |  |
| Marital status (ref. single) |  |  |  |  |  |  |  |  |
| Married |  |  | 0.046 | 0.028 | 0.033 | 0.02 | 0.066 | 0.04 |
|  |  |  | -0.055 |  | -0.055 |  | -0.061 |  |
| Cohabiting |  |  | -0.014 | -0.007 | -0.027 | -0.014 | -0.026 | -0.014 |
|  |  |  | -0.063 |  | -0.062 |  | -0.07 |  |
| Education (ref. primary) |  |  |  |  |  |  |  |  |
| Occupational secondary |  |  | -0.04 | -0.021 | -0.072 | -0.038 | -0.084 | -0.044 |
|  |  |  | -0.099 |  | -0.098 |  | -0.108 |  |
| General secondary |  |  | 0.198* | *0.121** | 0.147 | *0.09* | 0.124 | *0.075* |
|  |  |  | -0.094 |  | -0.094 |  | -0.103 |  |
| Tertiary |  |  | 0.294** | 0.163** | 0.212* | 0.118* | 0.178 | 0.098 |
|  |  |  | -0.098 |  | -0.099 |  | -0.109 |  |
| Economically active |  |  | 0.107* | 0.064* | -0.037 | -0.022 | -0.144* | -0.086* |
|  |  |  | -0.05 |  | -0.06 |  | -0.07 |  |
| Income (imputed) |  |  |  |  | 0.042** | 0.161** |  |  |
|  |  |  |  |  | -0.01 |  |  |  |
| Income |  |  |  |  |  |  | 0.055** | 0.238** |
|  |  |  |  |  |  |  | -0.01 |  |
| Constant | 3.728** |  | 3.471** |  | 3.419** |  | 3.559** |  |
|  | -0.074 |  | -0.106 |  | -0.106 |  | -0.114 |  |
| N | 1021 | | | | | | 809 | |
| R2 | 0.16 | | 0.19 | | 0.20 | | 0.24 | |
| Adj. R2 | 0.15 | | 0.18 | | 0.19 | | 0.23 | |
| BIC | 2316.1 | | 2319.4 | | 2307.2 | | 1813.1 | |

Standardized (beta) and unstandardized coefficients, standard errors, significance tests (t-test: * *p* < 0.05; ** *p* < 0.01), Bayesian information criterion (BIC), the proportion of explained variance (R^2^), and R^2^ adjusted for degrees of freedom.

Model 2 entered marital status, education, and economic activity. Marital status did not predict SRH at the 0.05 significance level. In contrast, education and economic activity were positively associated with SRH. Specifically, tertiary education was on average associated with a 0.3 shift in SRH compared to those with primary education. Economic activity was also positively linked to SRH, but its role was weaker than education (see the beta coefficients in Table 4). Importantly, education, economic activity, and marital status added only 3% of the explained variance to the model.

Models 3 and 4 integrated income into the equation. Model 3 used the full sample, and missing values in the income variable were replaced by the mean produced by the multiple imputations. The association between income and SRH was significant and positive. Importantly, once income was included in the model, the coefficient for economic activity ceased to be significant at the 0.05 level. Inspection of the beta coefficients also showed that income was the most important variable among the socioeconomic characteristics. However, the proportion of explained variance increased only moderately (2 percentage points). This might be explained by the decline in the importance of education and economic activity once income was incorporated into the model. Model 3 suggested that all socioeconomic characteristics contributed to around 5% of the explained variance.

Model 4 re-ran the analysis only for the subsample with non-missing income values (N = 809). In this subsample, the role of income seemed to be stronger and contributed to around an additional 6% of explained variance. Nevertheless, we needed to be cautious about such an interpretation, as the characteristics of the subsample with non-missing values differed from the full sample. Thus, 25 regression analyses with the 25 imputed values were estimated. This estimation produced 25 values of R2 with a mean of 0.20 and a standard deviation of 0.003 (95% CI: 0.198–0.201). Thus, it was very likely that the role of income was overestimated in the subsample with non-missing values.

Model 1 in Table A3 used the linear biomarker measures; Model 2 used the binary biomarker measures, indicating whether the biomarker was over the reference limit; and Model 3 used the binary measures adjusted for medication. All three models revealed that the levels of C-reactive protein and blood glucose and the LDL/HDL ratio were negatively associated with SRH. The LDL level was negatively linked to SRH only when a linear biomarker measure was used. The association between SRH and the triglycerides index was not significant at the *p* < 0.01 level in any of these models. Importantly, the relative importance of the biomarkers differed among all three models (see the standardized beta coefficients for biomarkers in Model 1 – 3). If the linear measure was used, the LDL/HDL ratio was the most important predictor of SRH, followed by C-reactive protein. In Models 2 and 3, CRP was the most important predictor of SRH. As for the explained variance, biomarkers added around 4% to the explained variance (see baseline Model 1 in Table A2).

**Table A3:** Results of the ordinary least squares (OLS) regression models with dependent variable SRH, displaying regression coefficients, standardized coefficients (beta), standard errors (in parentheses), and significance level. Source: QUALITAS 2016/2017 survey (N = 1021).

|  | M1 (Linear) | | M2 (Binary) | | M3 (Binary adj.) | | M4 | | M5 | |
| --- | --- | --- | --- | --- | --- | --- | --- | --- | --- | --- |
|  | Coef. | Beta | Coef. | Beta | Coef. | Beta | Coef. | Beta | Coef. | Beta |
| Age | -0.018** | -0.358** | -0.018** | -0.363** | -0.018** | -0.349** | -0.018** | -0.360** | -0.017** | -0.345** |
|  | *(-0.002)* |  | *(-0.002)* |  | *(-0.002)* |  | *(-0.001)* |  | *(-0.002)* |  |
| Male | 0.085 | 0.052 | 0.054 | 0.033 | 0.060 | 0.037 | 0.083 | 0.051 | 0.064 | 0.039 |
|  | *(-0.052)* |  | *(-0.049)* |  | *(-0.050)* |  | *(-0.048)* |  | *(-0.047)* |  |
| Location | 0.038 | 0.024 | 0.018 | 0.011 | 0.022 | 0.014 | 0.025 | 0.016 | 0.026 | 0.016 |
|  | *(-0.046)* |  | *(-0.047)* |  | *(-0.046)* |  | *(-0.046)* |  | *(-0.046)* |  |
| CRP | -0.025** | -0.124** | -0.222** | -0.101** | -0.025** | -0.124** |  |  |  |  |
|  | *(-0.006)* |  | *(-0.065)* |  | *(-0.006)* |  |  |  |  |  |
| Glucose | -0.059** | -0.084** | -0.186** | -0.090** |  |  |  |  |  |  |
|  | *(-0.021)* |  | *(-0.065)* |  |  |  |  |  |  |  |
| Glucose adj. |  |  |  |  | -0.176** | -0.086** |  |  |  |  |
|  |  |  |  |  | *(-0.064)* |  |  |  |  |  |
| TG - triglycerides | -0.015 | -0.020 | -0.045 | -0.021 | -0.018 | -0.024 |  |  |  |  |
|  | *(-0.025)* |  | *(-0.068)* |  | *(-0.025)* |  |  |  |  |  |
| LDL | 0.086* | 0.091* | 0.056 | 0.034 |  |  |  |  |  |  |
|  | *(-0.041)* |  | *(-0.054)* |  |  |  |  |  |  |  |
| LDL adj. |  |  |  |  | 0.044 | 0.027 |  |  |  |  |
|  |  |  |  |  | *(-0.059)* |  |  |  |  |  |
| LDL/HDL ratio | -0.139** | -0.145** | -0.135* | -0.071* | -0.083* | -0.087* |  |  |  |  |
|  | *(-0.047)* |  | *(-0.067)* |  | *(-0.039)* |  |  |  |  |  |
| # of biomarkers |  |  |  |  |  |  | -0.128** | -0.176** |  |  |
| > ref. limit |  |  |  |  |  |  | *(-0.022)* |  |  |  |
| # of biomarkers |  |  |  |  |  |  |  |  | -0.097** | -0.154** |
| > ref. limit adj. |  |  |  |  |  |  |  |  | *(-0.019)* |  |
| Constant | 4.073** |  | 3.734** |  | 3.930** |  | 3.730** |  | 3.720** |  |
|  | *(-0.136)* |  | *(-0.074)* |  | *(-0.097)* |  | *(-0.072)* |  | *(-0.073)* |  |
| R2 | 0.20 |  | 0.19 |  | 0.20 |  | 0.18 |  | 0.18 |  |
| Adjusted R2 | 0.19 |  | 0.18 |  | 0.19 |  | 0.18 |  | 0.17 |  |
| BIC | 2296.8 |  | 2313.0 |  | 2300.9 |  | 2288.8 |  | 2298.1 |  |

Standardized (beta) and unstandardized coefficients, standard errors, significance tests (t-test: * *p* < 0.05; ** *p* < 0.01), Bayesian information criterion (BIC), the proportion of explained variance (R^2^), and R^2^ adjusted for degrees of freedom.

CRP - C-reactive protein, TG – triglycerides, LDL – low density lipoprotein, HDL – high density lipoprotein

Models 4 and 5 replaced biomarkers with summary indicators indicating how many reference levels were crossed. Clearly, the greater the number of biomarkers over the reference limits, the worse health the respondents reported. This summary indicator added around 2%–3% to the explained variance.

Model 1 in Table A4 entered the number of conditions the respondents were diagnosed with and the number of drug classes they were treated with. Both measures were negatively related to SRH. An additional condition was associated with a 0.2 shift in SRH, and an additional drug class was associated with a 0.14 shift. The beta coefficient showed that the importance of medication status as an SRH indicator was equally important as age, followed closely by the number of medical conditions. A comparison with the baseline model (Model 1 in Table A2) demonstrated that the self-reported number of medical conditions and medications contributed 11% to the explained variance.

**Table A4:** Results of ordinary least squares (OLS) regression models with dependent variable SRH, displaying regression coefficients, standardized coefficients (beta), standard errors (in parentheses), and significance level. Source: QUALITAS 2016/2017 survey (N = 1021).

|  | M1 | | M2 | | M3 | | M4 | |
| --- | --- | --- | --- | --- | --- | --- | --- | --- |
|  | Coef. | Beta | Coef. | Beta | Coef. | Beta | Coef. | Beta |
| Age | -0.011** | -0.225** | -0.020** | -0.399** | -0.017** | -0.330** | -0.012** | -0.232** |
|  | (*-0.002)* |  | (*-0.001)* |  | (*-0.002)* |  | (*-0.002)* |  |
| Male | 0.003 | 0.002 | -0.056 | -0.034 | 0.074 | 0.045 | -0.030 | -0.019 |
|  | (*-0.044)* |  | (*-0.043)* |  | (*-0.047)* |  | (*-0.043)* |  |
| Location | 0.074 | 0.046 | 0.050 | 0.031 | 0.028 | 0.017 | 0.071 | 0.044 |
|  | (*-0.044)* |  | (*-0.043)* |  | (*-0.045)* |  | (*-0.040)* |  |
| Medication # | -0.145** | -0.229** |  |  |  |  | -0.111** | -0.176** |
|  | (*-0.020)* |  |  |  |  |  | (*-0.019)* |  |
| Diagnoses # | -0.223** | -0.210** |  |  |  |  | -0.151** | -0.142** |
|  | (*-0.033)* |  |  |  |  |  | (*-0.030)* |  |
| Depressive s. |  |  | -0.668** | -0.379** |  |  | -0.552** | -0.314** |
|  |  |  | (*-0.047)* |  |  |  | (*-0.045)* |  |
| BMI |  |  |  |  | -0.040** | -0.244** | -0.023** | -0.139** |
|  |  |  |  |  | (*-0.005)* |  | (*-0.004)* |  |
| Alcohol (0 - ref.) |  |  |  |  |  |  |  |  |
| 1 |  |  |  |  | 0.024 | 0.011 | 0.013 | 0.006 |
|  |  |  |  |  | (*-0.066)* |  | (*-0.058)* |  |
| 2-5 |  |  |  |  | 0.050 | 0.024 | 0.084 | 0.040 |
|  |  |  |  |  | (*-0.065)* |  | (*-0.057)* |  |
| 6+ |  |  |  |  | 0.002 | 0.001 | 0.023 | 0.008 |
|  |  |  |  |  | (*-0.087)* |  | (*-0.077)* |  |
| Smoker |  |  |  |  | -0.180** | -0.097** | -0.159** | -0.085** |
|  |  |  |  |  | (*-0.052)* |  | (*-0.046)* |  |
| Constant | 3.554** |  | 3.757** |  | 4.632** |  | 4.138** |  |
|  | (*-0.070)* |  | (*-0.067)* |  | (*-0.134)* |  | (*-0.124)* |  |
| R2 | 0.26 |  | 0.30 |  | 0.22 |  | 0.39 |  |
| Adjusted R2 | 0.26 |  | 0.29 |  | 0.21 |  | 0.38 |  |
| BIC | 2189.6 |  | 2135.5 |  | 2273.7 |  | 2041.1 |  |

Standardized (beta) and unstandardized coefficients, standard errors, significance tests (t-test: * *p* < 0.05; ** *p* < 0.01), Bayesian information criterion (BIC), the proportion of explained variance (R^2^), and R^2^ adjusted for degrees of freedom.

Diagnoses #: the number of conditions respondents was diagnosed with

Medication #: the number of medication groups respondents was treated with

Model 2 in Table A4 added the mental health index. It showed that mental health was also significantly linked to SRH, and it contributed 15% to R^2^ (see Model 2 in Table A4).

Model 3 integrated BMI and behavioral items. The association between alcohol consumption and SRH was not significant at the 0.05 level, while both BMI and smoking were negatively linked to SRH. However, the standardized coefficients showed that the importance of smoking was relatively weak. BMI had an approximately 2.5 times greater effect on SRH than smoking. As for the proportion of explained variance, BMI and behavioral items contributed 6% to R^2^.

The final model, shown in Table 6, included controls, the number of conditions respondents were diagnosed with, the number of drug classes they were treated with, mental health status, BMI, alcohol consumption, and smoking. Together, these variables contributed nearly 40% to explained variance (R^2^ = 0.39; adjusted R^2^ = 0.38).
