# Supplementary material for: Assessment of self-rated health: The relative importance of physiological, mental, and socioeconomic factors
Source: PLoS One. 2022 Apr 18;17(4):e0267115. doi: 10.1371/journal.pone.0267115 (PMC9015117; doi:10.1371/journal.pone.0267115)
Supplement: Attachment — Submitted filename: Response to reviewers.docx [file pone.0267115.s002.docx]

**CHANGES IN RESPONSE TO REVIEWS**

First of all, we would like to thanks all reviewers for their time spent on reviewing our manuscript and for the valuable and thoughtful comments towards improving our manuscript. Before we address these concerns, point by point, we would like to describe the main conceptual changes.

Both reviewers expressed concerns about the order, in which variables entered the analysis. They argued that the stepwise building of the model and the order of variables block might significantly affect the results. To address these concerns, we changed our analytical strategy. First, we analyze the blocks of variables separately only controlling for age, sex, and location. Second, we built the final model using these blocks. As suggested by Reviewer 1, we start with the sociodemographic variables, and biomedical measures are entered only afterward.

**Reviewer #1:**

The aim of this study is to determine the relative importance of physical health, mental health, and SES in SRH in a sample of adult respondents from the Czech Republic.
This submission was easy to read. I am really sorry to say, however, that there are major methodological weaknesses. The first contribution, the authors state, is to confirm the validity of SRH in this sample. It not entirely clear either what this means (what type of validity?), nor how exactly it was approached methodologically.

We specify that we measure concurrent validity of SRH, i.e. the extent to which this indicator correlates with established measures of health. We also specify the measure that we are interested in in the relative contribution of different types of predictors.

Moreover, it is my impression that Czech respondents were included in a number of pan-European surveys of population health where SRH was the focus. I am sorry – I don’t know this literature but a quick scholar google search brings up a number of papers. Perhaps they aren’t 100% relevant, but a brief literature review on SRH in Czech respondents would be helpful.

We added a brief literature review on the Czech Republic. There is a number of studies using SRH but the research on validity of SRH is limited.

The second aim was to determine the relative importance of the three factors (physical health, mental health, and SES). However, the authors did this by estimating nested OLS models and looked at the increases in R squared. There are a number of issues.
1. Quite concerning: the authors state in the abstract that “Biomarkers (C-reactive protein, blood glucose, triglyceride, low density lipoprotein, high density lipoprotein), self-reported information on medical conditions, and BMI explain 27% of total variance in SRH.” That’s plain wrong, I am sorry. The physical health indicators explain an additional 11% beyond the 16% explained by age and sex.

This section was completely changed based on the other comments from both reviewers. The abstract has been re-written.

2. Similarly: the authors conclude that [SES] “contributed to only around 2% of the explained variance.” This is also misleading: it’s 2% after including all measures of physical and mental health measures, which, one might hypothesize, are the mechanisms through which SES and SRH are linked, at least partly.

Again, the section has been re-written and formulation changed.

**3. The methods section raised numerous questions.**
a. What was the response rate? b. Were weights used in the analyses? c. Who is in this sample, and how does the sample differ from the Czech population overall? What is the age range in particular, given that age is, by far, the most ‘important’ covariate of SRH?

We added a new section with the description of the dataset. As we use quota sampling, it is not possible to determine response rates or use weights.

d. In Marital status, does “single” mean “never married” or “not married or cohabiting”?

We specify that “single” means “currently living without a partner”.

e. How appropriate is OLS for the analysis of SRH? Did the authors conduct any diagnostics?

We report diagnostics of the OLS in the Appendix. We also refer to existing studies using OLS to study SRH.

f. There appears to be a lot of missingness. First, the authors do not discuss it beyond noting 21% missing values on income. What was missingness on other variables? Then they lay out what appears an irregular set of steps to deal with the missingness, culminating with a nonstandard way of apparently hand-averaging 25 imputations and plugging these averages into the models. That does not appear any better than a random realization from multiply imputed datasets and fundamentally fails to account for the increased uncertainty due to the missingness. Perhaps I misunderstand – in that case, could the authors clarify the procedures?

In general, income was the only variable with a larger proportion of missing data. We acknowledge that using a simple mean of imputed values is problematic. Thus, we employ three methods to cope with the missing data. First, we estimate the results only for cases without missing information. Second, we employ multiple imputations and we use the mean value of the imputed income (as it was done in the original version of the paper). Third, we estimate the set of regression models with the imputed data and we report on the distribution of R2 values.

g. For the biomarkers, how were respondents on medication treated? That is, fasting blood glucose can be normal because it’s normal or because an individual is on diabetes medication. Same with triglycerides and statins or other cholesterol medications.

In the revised version of the paper, we included a new variable referring to medications. As for the biomarkers, we distinguished those below and over the reference limit. In the analysis, we used both crude and adjusted measures. The crude measure refers to the number of biomarkers over the reference limits. The adjusted measure considers whether the respondent takes medication for the given condition.

h. It would be good to add an ANOVA test for difference in biomarkers by SRH levels.
We added ANOVA test for differences in biomarkers in Table 2.

4. The mental health indicator is only the CES-D scale for psychological distress (used to measure depressive symptoms). While sleep quality is included, too, this is not a measure of mental health. Or perhaps, in absence of other indicators, it could serve as a proxy for some dimension of mental health but the authors do not explain or motivate this unusual indicator.

We adjusted the analysis. The original CES-D scale contains an item on sleep disturbances, but this item has not been included in the Qualitas questionnaire. Instead, it was replaced by a different measure of sleep quality. In the revised version of the paper, we run a measurement model (confirmatory factor analysis using SEM technique) to test whether our measure of sleep quality could be included in the measure of mental health. Indeed, the measurement model confirmed that all 4 items from CES-D and the sleep quality item load on one latent variable (mental health – the full measurement model is reported in the Appendix). Thus, in the revised version of the paper, we use one indicator of mental health including all 5 items.

5. Why is there no descriptive table, even in the Appendix?

We added a descriptive table (Table 1).

6. Given that the sample appears to be drawn from two locations, why is there no control for location?

All models now control for the location.

7. The Figure 1 is a clear and compelling way to present the results. However, why is education included as a linear covariate? Table 2 suggests its effects are nonlinear. And why is there no indication about how income was included?

Education is now included as a categorical variable. In the figure, we denote which variable is linear, binary, and categorical.

8. The Figure might be more informative if, instead of bivariate models, it was based on age-adjusted models (or age and sex).

We adjusted the results for age, sex, and location.

9. The analysis includes BMI and sleep quality, and classifies them as physical and mental health measures, respectively. There’s no indicator of smoking or alcohol use, which makes me wonder why these behaviors are not included. The authors may push back that they didn’t set out to study health behaviors, which would be fair. However, it might be helpful to discuss this apparent omission at least in the discussion section.

Alcohol intake and smoking are now incorporated into the analysis.

10. Why are SES measures included in the Table 2 last? That would be very important to justify. I would expect to first control for SES (after age and sex) as the fundamental determinant of the other health measures. If done that way, perhaps the increase in proportion explained would still be modest, but it’s important to show what it would be.

In the revised version of the paper, we use a different analytical strategy. We first enter the blocks of variables independently. In the final model, we started with the controls, SES, and then add other health measures.

**Reviewer #2:**

The current manuscript describes a study based on data from a survey of adults in the Czech Republic. The participants filled in a questionnaire and provided a blood sample. This enabled the researchers to study the predictors of self-rated health (SRH) status and compare the relative contribution of several types of predictors: biomedical, psychological, and social indicators. While the predictors of SRH have been investigated in many studies, only a few included biomarkers. Those mostly used analyses that did not yield an exact estimate of the amount of variance explain by each type of predictor. Thus, analyses based on linear regression models of data that includes biomarkers may provide a worthwhile contribution to this literature. However, there are several issues which the authors could better address.
First and foremost is the determination of the relative contribution of each type of predictors. The authors focus on the addition to the adjust R2 with each additional step. This is greatly affected by the order of entry of the variables into the model. The actual unique contribution of each set of variables in the final model differs from its contribution in the step when it was first entered and is not reported. There was no trial of a different order of entry into the model. Alternatively, better explain why this order of entry of predictors was chosen. Is the aim to determine what part of SRH reflects physiological markers and disease diagnoses and what part is related to psychological measures, social indicators, and demographics? Why was this order of variables chosen? Or is the aim to identify health inequalities – in which case this aim deserves a more detailed explanation. The analyses are interesting, relevant to the question asked and, as the authors wrote, not often reported in this way (in studies including biomarkers), yet could be better explained.

In the revised version of the paper, we adopted a new analytical strategy that uses a different order of entering variables into the model (see the description above)

Several additional issues:

The introduction briefly describes existing research on the topic. Despite the lack of a clear comparison between types of predictors in their contribution to SRH, there are several additional articles that included a range of predictors and could be cited, to provide a fuller picture of the existing literature. For example, Goldman et a. 2009 (doi:10.1016/S1047-2797(03)00077-2), Anreasson et al., 2013 (DOI: 10.1177/1359105311435428).

The articles are added to the literature review.

The study procedure is barely described – much information is missing on the way potential participants were identified, approached and recruited to the study, where they provided the data, and on response rates.

We added better description of survey and participants’ characteristics.

CES-D – the questionnaire originally included 20 items. Shorter 10- or 11-item versions are often used and have been validated. A four item version is not commonly used and it would be appropriated to add information supporting its validity.

In the Appendix, we report a measurement model (SEM) and we show that the 4-item scale has a very good internal consistency. We comment on this fact in the main body of the paper.

Table 2 – if the aim is to compare the contribution of different predictors, it makes more sense to report standardized regression coefficients.

Standardized coefficients are included in all tables.

Study limitations – the study is cross-sectional. Longitudinal data may reveal additional information; some well-known correlates of SRH were not included, for example, physical functioning, cognitive functioning, positive emotions.

We acknowledged this limitation in the study limitations.

Corrections
Please correct typos and language errors on lines 45, 58, 93, 137, 156 (+define the abbreviation for CRP), 160, 170, 171, 181, 235, 263, 282, 296, number of diagnoses (in Table 2).

The article has been edited by a professional service specializing in academic writing, all abbreviations are defined.

Correct author names in reference 9 (Leshem-Robinow et al.).

The name as it is printed on the publication is used.
