# Supplementary material for: Assessment of self-rated health: The relative importance of physiological, mental, and socioeconomic factors
Source: PLoS One. 2022 Apr 18;17(4):e0267115. doi: 10.1371/journal.pone.0267115 (PMC9015117; doi:10.1371/journal.pone.0267115)
Supplement: Attachment — Submitted filename: Response to reviewers.docx [file pone.0267115.s003.docx]

We would like to thank both reviewers for their time and helpful comments that helped to improve the manuscript. We hope that we address all points raised in the reviews.

Before we address each point in detail, we would like to describe the major changes to the paper. First, we revised the introductory part to make it more focused and to explain the research agenda more clearly. Second, we followed the advice of Reviewer 1 who suggested to move much of the results to the Appendix. Thus, the result section is more concise and we believe it is easier to follow. Third, we expanded the discussion and we point out the major implications of our findings.

**Reviewer #1:**

The authors were highly responsive to all comments and the extensive revision yielded a much stronger paper. I am sorry, however, to pose several residual questions.

Introduction last paragraph: what is “this social context” in line 83? And in lines 88-90, do the authors refer to Czech republic?

The sentence has been reformulated to make it clear that we talk about the Czech Republic.

Methods: I didn’t understand why the quota sampling invalidates the question about response rate. Presumably some individuals who were approach did not participate: what was the response rate?

The information on how many respondents from the originally selected sample is provided.

Approach: about the multiple imputation, page 6 lines 131-2. How or where is the distribution of the 25 R squareds reported?

The section is moved to the Appendix.

Minor: I would suggest referring to the two locations as “location,” not “region.”

The term region has been replaced by “location” in the whole text.

Results: the fact that only 2 out of >1,000 people assessed their health as “very bad” suggests that the SRH item, or the labels used for its 5 categories in the Czech language, capture something different than the English labels of excellent, very good, good, fair, or poor. This should be discussed as a limitation.

In the discussion, we point out that this study utilized the WHO version of the SRH item ranging from “very good” to “very bad” that is widely used in European surveys. This item has generally better discrimination power at the positive end.

Results: more importantly, the results are now much expanded but a bit difficult to follow. It seems the authors incorporated a lot of different approaches and robustness checks. I think the results section would be much stronger if they only retained one approach/specification and told the story with it; all the robustness checks could be in the appendix.

As suggested, we moved a significant part of the result section to the Appendix and we believe that the flow of the text is easier to follow in this version of the paper.

There is little discussion in general. One issue that I was hoping to see was to engage with the issue that socioeconomic factors are more distal characteristics, that may influence SRH via the more proximate factors, such as health conditions or biological risk variables.
More broadly, there is little discussion that would help readers understand the results. What do the findings mean for SRH, or for the Czech population health, or for population-health research? Why might it be that this study found such a weak relationship between SES and SRH? Etc.

We expanded the discussion in the suggested direction.

**Reviewer #3:**

The study examines associations of physiological, mental and socioeconomic factors with self-rated health, specifically “the relative contribution of biomedical and social characteristics to an individual´s SRH assessment” in a population sample from the Czech republic. The topic is basically relevant. Yet the paper has a major flaw in that the basic idea of the study seems to be missing. It remains unclear why these associations are important to know, why they are analyzed here, and what should we learn from the results.

We revised the introductory part of the paper and we hope that the basic idea of the study is expressed better. We try to explain as to why it is relevant to validate the SRH item in different contexts.

It is striking that the crucial part in all studies, interpretation of findings, is largely missing. The Discussion only repeats the statistical results and discusses limitations, concerning mainly data.

The results section is completely revised. Much of the detailed findings have been moved to the appendix. We also completely revised the discussion to make explain the contribution and implications of the study.

There are several starts in the Introduction that let the reader believe that this is may be the theme of the study, the perspective from which the associations/ relative explanatory powers are studied, but none of these themes seems to have guided the analyses, nor are they discussed in Discussion. The validity of SRH is mentioned in several places in the paper. Yet is remains unclear, what validity the authors have in their minds; the validity of SRH as a measure of objective health?; its validity as predictor of death?, or something else. …..

We completely revised the introductory section and we hope that the aim of the manuscript and the issue of validity is clearer.

If the authors decide to continue their work on this paper and maintain the focus on the explanatory power on different variable groups, they may also consider the following comments:
• It is problematic to claim (p3) that ”what contributes to an individual´s assessment of SRH remains largely unknown”. …

The section is revised.

• As the authors speak about the relative contribution of biomedical and social characteristics, I am sure many readers expect to learn about the relative contribution of these variable groups. Yet the biomedical and social variables are not analyzed as groups but only as individual variables.

We explain our approach in the method section. Our work is inspired by Hiyoshi et al.’s approach to estimating the joint contribution of several variables. Thus, we do not focus on individual coefficients but the joint contribution to the explained variance.

As such, the empirical findings are not novel. The authors write (“p4 ..”no study has tested the concurrent validity of SRH, ie the extent to which this indicator correlates with established measures of health, using biomedical data”. This is clearly wrong. ….

Here, we refer to the national context. Hopefully, it is clearer in the revised version.

• To an international audience it is not a very good justification of a study that these associations have not earlier been studied in a Czech population. Numerous studies show that SRH as a variable behaves largely in the same way in different countries, different population groups etc. If there is no good reason to believe that in Czech the association would be different, the justification of the study should be based on its scientific novelty and message. Of course, Czech population can work as well as any other for this purpose.

We hope that we explain the contribution of the manuscript clearer.
