# Supplementary material for: Assessment of self-rated health: The relative importance of physiological, mental, and socioeconomic factors
Source: PLoS One. 2022 Apr 18;17(4):e0267115. doi: 10.1371/journal.pone.0267115 (PMC9015117; doi:10.1371/journal.pone.0267115)
Supplement: Attachment — Submitted filename: Response to Reviews.docx [file pone.0267115.s004.docx]

P 4 says that face-to-face interviews were conducted, later a questionnaire is mentioned several times. Please clarify.

**We clarify that a questionnaire was administered by face-to-face mode of data collection.**

Due to sampling method, response rate could not be calculated.

**The comment on response rate was included as a reaction to a reviewer comment. It is deleted from the revised manuscript.**

In all likelihood the sample is not representative for the population in the study areas. This does not hamper the results but would be worth mentioning in Discussion.

**It is mentioned in the discussion.**

The limitation
A major well-known factor associated with SRH was missing, functioning, functional ability, disability... It would be good to mention this in the Discussion.

**It is mentioned in the discussion among the study limitations.**
